# Facebook Users’ Interactions, Organic Reach, and Engagement in a Smoking Cessation Intervention: Content Analysis

**DOI:** 10.2196/27853

**Published:** 2021-06-21

**Authors:** Dávid Pócs, Otília Adamovits, Jezdancher Watti, Róbert Kovács, Oguz Kelemen

**Affiliations:** 1 Department of Behavioral Sciences Faculty of Medicine University of Szeged Szeged Hungary

**Keywords:** smoking, smoking cessation, behavior, health behavior, internet, social media, love, comment, motivation, language, public health

## Abstract

**Background:**

Facebook can be a suitable platform for public health interventions. Facebook users can express their reaction to the given social media content in many ways using interaction buttons. The analysis of these interactions can be advantageous in increasing reach and engagement of public health interventions.

**Objective:**

This research aimed at understanding how Facebook users’ interactions correlate with organic reach and engagement regarding the same smoking cessation support contents.

**Methods:**

The study population consisted of Facebook users who were reached by a public smoking cessation support page without advertising. We included 1025 nonpaid Facebook posts (N=1025) which used smoking cessation strategies based on a motivational interviewing counseling style. The following data were collected from the “Post Details”: the number of people who saw the given nonpaid content (organic reach) which consisted of fan and nonfan reach according to previous “page like” activity; each rate of “engagement indicators” (such as the symbols of “like,” “love,” “haha,” “wow,” “sad,” “angry”; or other interactions: “shares,” “comments,” “clicks”); and the rate of negative Facebook interactions (eg, “post hides” or “unlike of page”). Overall, these data were analyzed with the Spearman correlation method.

**Results:**

Surprisingly, we found a significant negative correlation between organic reach and the “like” reaction (r_s_=–0.418; *P*<.001). The strongest significant positive correlations of organic reach were observed with the “haha” reaction (r_s_=0.396; *P*<.001), comments (r_s_=0.368; *P*<.001), and the “love” reaction (r_s_=0.264; *P*<.001). Furthermore, nonfan reach correlated positively with “shares” (r_s_=0.388; *P*<.001) and clicks (r_s_=0.135; *P*<.001), while fan reach correlated positively with the “haha” reaction (r_s_=0.457; *P*<.001), comments (r_s_=0.393; *P*<.001), and the “love” reaction (r_s_=0.310; *P*<.001). Contrary to expectations, the “like” reaction was sharply separated by significant negative correlations from “wow” (r_s_=–0.077; *P*=.013), “sad” (r_s_=–0.120; *P*<.001), “angry” reactions (r_s_=–0.136; *P*<.001), and comments (r_s_=–0.130; *P*<.001). Additionally, a high rate of negative Facebook interactions was significantly associated with “wow” (r_s_=0.076; *P*=.016) and “sad” reactions (r_s_=0.091; *P*=.003).

**Conclusions:**

This study has shown that it is possible to hypothesize a disadvantage of the “like” reaction and advantages of other interactions (eg, the “haha” reaction or “comments”) in content algorithmic ranking on Facebook. In addition, the correlational analysis revealed a need of a further categorization to fan-specific interactions (eg, “haha” or “love” reactions) and nonfan-specific interactions (eg, “shares” and “clicks”). Regarding the direction of the correlations, these findings suggest that some interactions (eg, negative Facebook interactions, “wow,” “sad,” and “angry” reactions) may decrease the engagement, while other interactions (“like,” “love,” “haha” reactions, “shares,” and “clicks”) may increase the engagement during Facebook-based smoking cessation interventions. This hypothesis-generating research offers an important insight into the relationship between organic reach, engagement, and Facebook users’ interactions for public health professionals who design Facebook-based interventions.

## Introduction

### Reaching to Facebook Users

Facebook is a widely available social media platform, which could be highly relevant for people who seek help with health behavior change [1]. Facebook is also used by public health organizations to communicate health messages [2,3], especially for reducing smoking [4-7]. Social media contents which support smoking cessation can be more cost-effective than television advertising [8]. Facebook can also be a useful tool to contact hard-to-reach smokers [9]. On this platform a major intention of smoking cessation or other public health interventions could be to reach a large number of users through social media contents [10]. A commonly used measure of dissemination can be the Facebook “post reach” data, which represent the number of people who saw the given social media content [11,12]. Facebook provides access to these reach data and allows page administrators to increase the post reach by paying [13]. Therefore, “paid post reach” and “organic post reach” data must be distinguished. These data refer to the number of people who saw a paid or a nonpaid social media content, respectively [11,14]. Previous research has found that nonpaid (organic) reach is associated with higher engagement than paid reach [11]. “Post reach” data can also be divided into fan reach and nonfan reach based on the previous usage of the “page like” button.

An increasing number of contents are being published on Facebook; however, the number of posts a Facebook user is able to see at a time is limited. Therefore, Facebook must filter the social media contents for the users, and contents compete with each other to reach Facebook users [15,16]. This phenomenon has opened an exciting direction for research to reveal the opportunities to increase organic post reach in Facebook-based public health interventions [17,18]. Because of the increasing number of posts, Facebook provides only the most relevant content to each user [15,18]. The basis of this highly personalized filtering is the “Facebook algorithm of content ranking,” which ranks all available posts that can be displayed on a user’s News Feed. This algorithm is reviewed annually, but the details of the Facebook algorithm are unknown (ie, not published) [11,15]. However, some major elements of the algorithm which may determine the Facebook News Feed content are suspected, such as the Facebook user’s past activity (eg, sending a message to the page or using the “page like” button); the past activity of the Facebook page (eg, violating the Facebook Community Standards); the performance of the given post (the rate of “like,” “shares,” “clicks,” or other interactions); the post type (eg, image or video); or the timing of the published content (eg, novelty) [2,11,16-18].

These indicate a need to understand how to correlate Facebook users’ interactions with organic (fan and nonfan) reach during a smoking cessation intervention, which is a major aim of our research. The platform of the investigated smoking cessation intervention was a public Facebook page, which published social media contents based on a motivational interviewing counseling style. We have summarized our problem statements, aims, and research questions regarding organic reach in Textbox 1. The relationship between total organic reach and different Facebook interactions on a post level may highlight the way in which the Facebook algorithm ranks available contents according to Facebook users’ interactions. For example, some interactions can have a higher impact on content algorithmic ranking due to stronger positive correlation with organic reach, while other interactions can have a negative role in content ranking because of negative correlation with organic reach. Likewise, the association between fan reach, nonfan reach, and Facebook interactions may show which element is preferred by the algorithm: for example, Facebook users’ interactions on the given post or previous “page like” activity. For instance, some interactions, which correlate negatively with fan reach, can have a higher impact on content ranking than previous “page like.” Finally, the correlational analysis between organic reach and Facebook interactions separately for each year may also show how the Facebook algorithm of content ranking is modified annually. This study is an exploratory research, which analyses a set of data searching for correlations, and then proposes hypotheses which may then be tested in subsequent studies.

### Interactions on Facebook

A Facebook interaction on a post level is defined as any action on specific buttons that the user performs in relation to content, and it can be divided into 3 groups: “positive interactions” (reactions, shares, comments), “neutral interactions” (clicks), and “negative interactions” (post hides, hides of all posts, reports of spam, unlike of page) [2,11,19-22]. The Facebook reactions (such as like, love, haha, wow, sad, angry) are designed to give users a more nuanced way to express their emotions, and these could have a special role in public health interventions [22]. For example, a tobacco cessation study has shown that receiving 1 “like” reaction on the Facebook-based intervention platform is associated with smoking reduction by approximately 1 cigarette per week [23]. The “like” reaction was introduced in 2009, while other reactions were instituted in 2016, so there has been little quantitative analysis of “love,” “haha,” “wow,” “sad,” or “angry” reactions in public health interventions so far [2,22].

The “share” interaction covers several ways of sending the content with optional privacy settings to others, such as share content on the user’s or a friend’s timeline, share in a Facebook group, share to a Facebook page, or send as a message via Facebook [24,25]. Internet users tend to share content when they perceive a low risk of reputational damage and high benefits to their social network; nevertheless, further investigation is needed to find what makes public health content be shared [11,26]. With the “comment” button, users can publish a text or an image message under a given Facebook post [26,27]. The total number of clicks contains more neutral actions on the content, such as selecting a website, viewing the Facebook page profile, or expanding photos to full screen [28].

Facebook users’ interactions can be placed in a theoretical framework of “engagement” [29]. In previous behavioral science studies, “engagement” of digital behavior change interventions was interpreted as the extent of usage [30,31]. In Facebook-based interventions, engagement can be defined as a group of Facebook interactions. These “engagement indicators” include “reactions,” “shares,” “comments,” and “clicks” [11,12,32]. Several attempts have been made to determine the “depth” or the “spectrum” of engagement on the basis of the fact that some Facebook interactions (eg, “shares”) can have higher engagement than others (eg, the “like” reaction) [33]. However, little is known about the spectrum of engagement, and it is still not clear which Facebook interactions may stand at a similar engagement level, or which interactions may occur as a combination of engagement indicators [29,33]. Furthermore, the spectrum of engagement raises a need to understand the relationship between positive emotional interactions (eg, “like” or “love” reactions) and negative emotional interactions (eg, “sad” or “angry” reactions). As opposed to engagement, negative Facebook interactions (post hides, hides of all posts, reports of spam, unlike of page) could indicate a neglect of the Facebook-based intervention as a negative engagement indicator. Nevertheless, there has been little discussion in the literature about the role of negative Facebook interactions in online public health interventions. Textbox 1 provides the problem statements, aims, and research questions related to engagement.

**Problem statements, aims, and research questions of this study.**

**Problem Statement 1**

Broad reach is an essential condition for a successful web-based public health intervention. Organic reach on the Facebook platform depends on a hidden algorithm, which includes Facebook users’ interactions.

**Aim**

To understand the association between organic reach and Facebook interactions during a smoking cessation intervention.

**Research Questions**

- What is the relationship between total organic reach and Facebook interactions on a post level during a smoking cessation intervention?
- How fan reach and nonfan reach of identical intervention contents are influenced by different Facebook interactions?
- How do the correlations between total organic reach and Facebook interactions change each year (in parallel with a modified Facebook algorithm)?

**Problem Statement 2**

High engagement rate is a relevant feature for a successful web-based public health intervention. Engagement on Facebook can be defined as a combination of interactions. However, some Facebook interactions may express positive emotions, while others may express negative emotions.

**Aim**

To assess the correlations between positive and negative emotional interactions during a Facebook-based smoking cessation intervention.

**Research Questions**

- How does the direction of correlations change between engagement indicators which express positive and negative emotion during a smoking cessation intervention?
- How does the strength of positive correlations change between engagement indicators at similar smoking cessation support contents?
- What is the relationship between engagement indicators and negative Facebook interactions on a post level during a smoking cessation intervention?

## Methods

### Stimuli

The investigated Hungarian “Cigarette break” Facebook page [34] was a nonbusiness and nongovernmental smoking cessation intervention. The primary aim of this program was to avoid frightening and judgmental communication about smoking, and support smoking cessation using a motivational interviewing counseling style (ie, building on collaboration, partnership, and empathetic understanding; emphasizing the autonomy of smokers; supporting self-efficacy). Secondary aims of the intervention were harm reduction in smokers, relapse prevention in former smokers, and developing assertive and social support skills in nonsmokers. Motivational interviewing strategies have been deliberately involved in the moderator work and the creation of Facebook posts. Social media contents were usually published daily at 5 pm on weekdays and at 2 pm on weekends from the beginning (March 7, 2017). The page was edited and managed by the authors, university students, and health care professionals experienced in motivational interviewing.

In all, 1269 social media contents were made during the research period, between March 7, 2017 and August 14, 2020. We excluded 244 Facebook posts in accordance with the following exclusion criteria. First, we excluded 60 “boosted” Facebook posts, which were promoted by paid Facebook advertising after publication to increase reach and engagement. Boosted posts can influence the results through the rate of interactions [11,33]. They can reach more Facebook users who are active in engagement before the advertising, rather than other passive users. Second, 24 video posts were also excluded because the evaluation of the interactions was fundamentally different from the assessment of the image posts (eg, minutes viewed, 10-second views), and also because the Facebook algorithm evaluates these posts differently during content ranking [2,16]. Third, we excluded 69 administrator’s posts and 16 posts which were targeted at nonsmokers. We only included contents that directly addressed to smokers. Fourth, 68 social media contents were excluded because of noncessation topic (eg, second-hand smoking or harm reduction). Only smoking cessation support Facebook posts were included to focus on public health interventions. Finally, we excluded 7 motivational interviewing nonadherent posts, which did not conform perfectly to the spirit of motivational interviewing during the retrospective analysis. It should be emphasized that only original Facebook posts were analyzed to evaluate the users’ response given to the same stimulus. Therefore, shared Facebook posts were ignored, because in these cases, Facebook users’ responses could have been influenced by other stimuli (eg, the Facebook profile of the person who shared the content), which may have resulted in a higher rate of interactions than the original content [18].

After content exclusion, 1025 original posts were included, which all followed the spirit of motivational interviewing, supported smoking cessation, were targeted at smokers, and did not use specific advertising. The majority of the social media contents included (994/1025, 96.97) were image posts, but some (31/1025, 3.02%) had only texts and links to other public health websites. The stimuli were smoking cessation support contents based on motivational interviewing on a public Facebook page; therefore, the content analysis can only be interpreted in this context. We present some examples of motivational interviewing style contents (stimuli) in Multimedia Appendix 1 to illustrate generalizability of the findings to other Facebook-based smoking cessation interventions. We also show some examples of excluded contents in Multimedia Appendix 2.

It should be highlighted that these social media contents were public, and Facebook users used different interaction opportunities voluntarily and freely, without external coercion. It should be also noted that our researcher identity is transparent to anyone in the description of the Facebook page. In addition, we provided information about the research and results through our publicly available posts.

### Participants

The theoretical population of this research was formed by any Facebook user who was reached by the included, nonpaid contents of the investigated public Facebook page. It is difficult to precisely determine the study population because the Facebook platform and the audience of the public Facebook page could have changed during this long research period. Therefore, we used a convenience sample. However, Facebook users’ data on a page level were exported from “Facebook Insights” on August 14, 2020, which contains epidemiological characteristics.

It should be emphasized that by creating Facebook profiles, users accept the terms and conditions of Facebook. These terms indicate that their data may be accessed by a third party. With this informed consent, Facebook provides anonymized and aggregate data to the page administrators.

At that time, the investigated Facebook page had 10,098 “Facebook fans,” who expressed their interest in and support for the page by a “Facebook page like.” Based on the age and gender information they provided in their user profiles, 52.91% of the Facebook page fans (5343/10,098) were women, and 46.97% (4744/10,098) were men, and 83.4% (8457/10,098) were between the ages of 18 and 34 years. The vast majority (9634/10,098, 95.40%) were from Hungary; but some German (96/10,098, 0.5%), Serbian (79/10,098, 0.78%), and Romanian (78/10,098, 0.77%) Facebook locations also presented; 9720/10,098 (96.25%) Facebook fans spoke Hungarian, 275/10,098 (2.72%) English, and 103/10,098 (1.02) other languages, based on the default language setting selected.

By contrast, the monthly total reach of the Facebook page was wider: 96,654 people saw the Facebook page’s contents in the last month; 43% of them were women and 57% were men; and 85% were between the ages of 18 and 34 years. The distribution of location and language data was roughly similar to the Facebook fans’ characteristics. The monthly total reach consisted of paid reach (66,463 people) and organic reach (30,191 people). Unfortunately, Facebook does not register users’ smoking status, therefore the smoking habits of the target population were unknown. However, we surveyed smoking status among the audience of this Facebook page in our previous study, where we found 65% were current smokers, 12% former smokers, and 23% nonsmokers [35]. The majority of the smokers (94%) used tobacco daily, while the minority (6%) used tobacco occasionally [35]. The most common nicotine product used in the sample was cigarette (98%), followed by e-cigarette (32%), hookah (20%), cigar (6%), snus (2%), pipe (2%), and snuff (2%) [35].

### Design

Our research method was a hypothesis-generating, retrospective, quantitative content analysis. The Facebook posts’ organic reach and interaction data were analyzed with the Spearman correlation method. These data were collected from the “Post Details” and belonged to the same social media content (same stimulus). Therefore, we analyzed data on a post level.

It should be noted that in our research we analyzed only 2 well-known elements of the Facebook algorithmic content ranking: users’ interactions on a post level (performance of the given post) and previous “page like” through fan reach (Facebook user’s past activity). There was not a remarkable difference in other known elements of the Facebook algorithm: mostly image contents were included (post type), all contents were regularly published at the same time (timing of published content), and the management of the investigated Facebook page was not changed notably (past activity of the Facebook page).

The definitions of organic reach and the different types of interactions were discussed in the “Introduction” section and we summarize them in Textbox 2. Facebook interactions can indicate how social media contents increase the usage of a Facebook-based intervention (reactions, shares, comments, clicks) or decrease it (negative Facebook interactions). We used the total number of negative Facebook interactions during the analysis, because it was available together and not separate in “Post Details.” It should be noted that the combination of interactions (eg, the “like” reaction and the “share” interaction) could arrive from different users or the same Facebook user, because these data were summarized. However, the combination of reactions on the same content (eg, “like” and “love” reactions) indicates different Facebook users, because 1 Facebook user could choose only 1 reaction. It should be emphasized again that organic reach and Facebook interaction data were anonymized and aggregated, so Facebook users could not be identified.

Facebook uses a private algorithm for highly personalized filtering of social media contents, which influences the methodology of our research at 2 points. First, the correlation between organic reach and interactions were also analyzed separately by years because Facebook changes this algorithm annually. Second, interaction data had to be corrected for the organic reach: the number of each interaction was divided by the number of people who saw the nonpaid post (organic reach) for the statistical analysis. This correction was necessary because increased organic reach can directly enhance other interaction numbers. In other words, if more Facebook users see the post, they are more likely to use interaction buttons. Consequently, the correlational analysis between organic reach and the total number of Facebook interactions would highlight a simple relationship rather than the impact of the Facebook algorithmic content ranking. However, we used an “interaction rate” to express the frequency of the given interaction at the same organic reach. Facebook uses the same correction of interaction data (called “engagement rate”), which is the number of people who liked, commented, shared, or clicked on the post divided by organic reach. “Engagement rate” can be accessed by page managers in the “Facebook Insights,” and the Facebook algorithm of content ranking may use this rate (as a performance indicator of the given post). Therefore, the “interaction rate” which was used in this study can be advantageous for the correlational analysis between organic reach and Facebook interactions or between Facebook interactions.

**Definitions of organic reach, different interactions, and engagement used in the current research.**

**Organic Reach**

The number of people who saw the given nonpaid social media content.

- *Fan reach*: The number of people who had liked the Facebook page before they saw the given nonpaid social media content.
- *Nonfan reach*: The number of people who had not liked the Facebook page before they saw the given nonpaid social media content.

**Facebook Interactions**

Any action on specific buttons that the user performs in relation to content.

- *Reactions*: The number of people who used a “like,” “love,” “haha,” “wow,” “sad,” or “angry” reaction button under a given social media content to express their emotions.
- *Share*: The number of people who used the “share” button under a given social media content to send the content with optional privacy settings to others.
- *Comment*: The number of people who used the “comment” button under a given social media content to publish a text or an image message.
- *Click*: The number of people who used any other actions on a given social media content, for example, to select a website, to view the Facebook page profile, or to expand photos to full screen.
- *Negative Facebook interactions*: The total number of people who used the following functions: post hides, hides of all posts, reports of spam, unlike of page.

**Engagement**

A group of the following Facebook interactions: reactions, share, comment, and clicks. These interactions are called *engagement indicators* and have been defined previously.

### Procedure

Our retrospective research aim was to analyze the data of Facebook posts published between March 7, 2017, and August 14, 2020. Two weeks were provided between August 14 and August 28 for the internet users to give a response to the last investigated Facebook post. The data collection was performed between August 28, 2020, and August 30, 2020. First, 1025 Facebook posts were included based on our exclusion criteria (N=1025). Second, organic reach and Facebook interaction data were collected from the “Post Details.” Third, we divided interaction data with organic reach to achieve “interaction rate.” Then, we assessed Spearman correlation between organic reach and Facebook interactions in the research period. In a second analysis, this correlation was investigated separately for each year to evaluate the potential effect of the Facebook algorithm which is modified annually. Finally, we also used Spearman correlation between Facebook interactions.

All analyses were conducted using the SPSS software (IBM). Correlation statistics were performed rather than regression because the cause-and-effect relationship between the investigated variables was unclear. We used the nonparametric Spearman correlation, rather than the Pearson correlation, because of the non-normal distribution of the data. In all analyses, the conditions of the Spearman correlation were met; variables were measured on an interval or ratio scale, variables represented paired observations, and monotonic relationship was detected between the variables using scatterplot test. The *P* value of less than .05 was taken to indicate a significant effect, and the *P* value of less than .001 was taken to indicate a highly significant effect. Original data supporting the results presented in the paper can be found in Multimedia Appendix 3. The data on statistical analyses are available from the corresponding author upon request.

## Results

### Trends in Reach and Interactions

We summarized the mean and SD of organic reach and Facebook interactions in Table 1. Organic reach is divided into fan and nonfan columns to illustrate the distribution of the average (total) organic reach between these 2 indicators. The average number of organic reach in the research period was 1328 (SD 981), of which 59% were Facebook page fan (783 Facebook users) and 41% were nonfan participants (545 Facebook users). Table 1 shows that the organic reach of social media contents gradually increased from one year to another in parallel with a growing fan reach percentage.

In the research period, the average number of Facebook interactions for 1 social media content was 15.8 “like” reaction (SD 11.7), 0.9 “love” reaction (SD 4.3), 3.2 “haha” reaction (SD 7.3), 0.3 “wow” reaction (SD 2.2), 0.2 “sad” reaction (SD 0.8), 0.1 “angry” reaction (SD 1.3), 2.4 “share” interaction (SD 3.7), 3.2 “comment” interaction (SD 7.1), 84.9 “click” interaction (SD 163.8), and 0.1 negative interaction (SD 0.4). It should be highlighted that the low number of “sad,” “angry” reactions, and negative interactions can be attributed to the spirit of motivational interviewing (eg, partnership or acceptance), which was a primary consideration in the creation of the investigated Facebook posts. Table 1 represents that some interactions (“haha” and “love” reactions or comments) followed the notable increase in organic reach from year to year. By contrast, the mean of some interactions (shares or negative Facebook interactions) did not change remarkably annually. Therefore, despite the greater number of Facebook users who saw the social media content, the activity of Facebook users regarding shares and negative Facebook interactions probably decreased.

**Table 1:** Mean and SD of organic reach and Facebook interactions in the research period together and separately for each year.

Period	Organic reach	Facebook interactions: Engagement indicators, mean (SD)													
		Reaction							Share		Comment		Click		Negative interactions
		Like	Love	Haha	Wow	Sad	Angry								
ALL^a^ (N=1025)^b^	1328.30 (981.23)	15.84 (11.7)	0.91 (4.29)	3.17 (7.33)	0.31 (2.20)	0.18 (0.84)	0.11 (1.29)	2.39 (3.68)		3.17 (7.06)		84.89 (163.82)		0.14 (0.40)	
2017 (N=215)^c^	709.38 (565.02)	11.78 (6.90)	0.15 (0.99)	0.48 (1.41)	0.08 (0.32)	0.10 (0.63)	0.03 (0.29)	1.67 (2.64)		1.31 (3.40)		70.36 (89.93)		0.14 (0.39)	
2018 (N=378)^d^	1380.96 (1107.79)	16.39 (11.4)	0.39 (1.64)	2.73 (5.27)	0.17 (1.98)	0.10 (0.45)	0.10 (0.65)	3.07 (5.00)		3.14 (6.44)		92.30 (216.93)		0.08 (0.32)	
2019 (N=299)^e^	1518.32 (775.13)	17.92 (14.2)	1.08 (4.27)	4.58 (8.58)	0.43 (2.72)	0.25 (1.17)	0.07 (0.50)	2.31 (2.48)		3.65 (7.32)		73.31 (97.33)		0.19 (0.46)	
2020 (N=133)^f^	1752.00 (1093.45)	16.19 (10.7)	3.22 (9.23)	5.58 (12.08)	0.75 (3.01)	0.35 (1.05)	0.36 (3.31)	1.76 (2.45)		5.17 (10.90)		113.36 (194.08)		0.17 (0.44)	

^a^All contents in the research period (2017-2020).

^b^Fan and nonfan reach: 59% and 41%, respectively.

^c^Fan and nonfan reach: 38% and 62%, respectively.

^d^Fan and nonfan reach: 55% and 45%, respectively.

^e^Fan and nonfan reach: 67% and 33%, respectively.

^f^Fan and nonfan reach: 65% and 35%, respectively.

### Correlations Between Reach and Interactions

The results of the correlational analysis between organic reach and Facebook interactions are summarized in Table 2. First, we analyzed the organic reach for the research period. The first question in this research area was, “What is the relationship between total organic reach and Facebook interactions?” We found that most of the interactions had positive correlations with total organic reach, except the “like” reaction and the “share” interaction. The most surprising result was a highly significant negative correlation between the “like” reaction and organic reach (*P*<.001). Furthermore, we could not find a significant difference between the “share” interaction and total organic reach in the research period, although the correlational analysis was nearly significant (*P*=.057). In addition, the highly significant positive correlation between negative interactions and organic reach was interesting (*P*<.001). The strongest highly significant positive correlations were observed between total organic reach and the “haha” reaction (r_s_=0.396, *P*<.001), comments (r_s_=0.368, *P*<.001), and the “love” reaction (r_s_=0.264, *P*<.001). In summary, these results suggest that the Facebook algorithm of content ranking took the interactions into account to varying degrees for the calculation of total organic reach.

The second research question was, “How are fan reach and nonfan reach of the investigated contents influenced by different Facebook interactions?” The direction of significant fan reach and nonfan reach correlations was the same for most interactions, except for the “share” and “click” interactions. We found an opposite direction of 2 highly significant correlations between the “share” interaction and fan reach (r_s_=–0.177; *P*<.001), and between the “share” interaction and nonfan reach (r_s_=0.388; *P*<.001). This result may explain the absence of a significant relationship between the “share” interaction and total organic reach. Furthermore, a highly significant positive correlation was found between “clicks” and nonfan reach (*P*<.001), while there was no significant relationship between “clicks” and fan reach (*P*=.87). Therefore, social media contents which generated a high rate of “shares” and “clicks” reached relatively more nonfan Facebook users than fan Facebook users. By contrast, the strongest highly significant positive correlations were observed between fan reach and the “haha” reaction (r_s_=0.457, *P*<.001), comments (r_s_=0.393, *P*<.001), and the “love” reaction (r_s_=0.310, *P*<.001). By contrast, less strong correlations were found between nonfan reach and the “haha” reaction (r_s_=0.153, *P*<.001), comments (r_s_=0.192, *P*<.001), and the “love” reaction (r_s_=0.135, *P*<001). These results suggest that social media contents which evoked a high rate of “haha” and “love” reactions and “comments” reached relatively more fan Facebook users than nonfan Facebook users.

Second, the Spearman correlation between organic reach and Facebook interactions was tested separately for each year in the research period. The last question in this research area was, “What are the correlations between organic reach and Facebook interactions each year?” The most striking result to emerge from the data is that the negative correlation between total organic reach and the “like” reaction gradually weakened, while positive correlations between total organic reach and some interactions (“haha,” “wow,” “sad,” “angry” reactions, comments, clicks) strengthened year by year. Similar observations can be made for the fan reach and nonfan reach. Taken together, these results might reflect the modifications to the Facebook algorithmic content ranking during the research period, if we assume that the other elements of the Facebook algorithm did not change significantly in those years.

**Table 2:** Spearman correlation between Facebook interactions and organic reach in the research period together and separately for each year.

Organic reach		Facebook interactions: Engagement indicators (Spearman correlation coefficients)									
		Reaction						Share	Comment	Click	Negative interactions
		Like	Love	Haha	Wow	Sad	Angry				
**ALL^a^ (N=1025)**											
	Total	–0.418^b^	0.264^b^	0.396^b^	0.164^b^	0.231^b^	0.160^b^	0.059	0.368^b^	0.076^c^	0.124^b^
	Fan	–0.352^b^	0.310^b^	0.457^b^	0.169^b^	0.245^b^	0.173^b^	–0.174^b^	0.393^b^	–0.005	0.089^c^
	Nonfan	–0.332^b^	0.135^b^	0.153^b^	0.096^c^	0.144^b^	0.103^c^	0.388^b^	0.192^b^	0.135^b^	0.104^c^
**2017 (N=215)**											
	Total	–0.633^b^	0.028	0.142^c^	0.092	0.113	0.091	0.255^b^	0.266^b^	0.124	0.240^b^
	Fan	–0.412^b^	–0.013	0.144^c^	0.086	0.166^c^	0.063	–0.186^c^	0.234^c^	0.147^c^	0.144^c^
	Nonfan	–0.549^b^	0.056	0.011	0.071	0.033	0.069	0.392^b^	0.195^c^	0.092	0.196^c^
**2018 (N=378)**											
	Total	–0.296^b^	0.178^c^	0.295^b^	0.137^c^	0.180^b^	0.152^c^	0.157^c^	0.403^b^	0.305^b^	0.013
	Fan	–0.202^b^	0.173^c^	0.420^b^	0.140^c^	0.236^b^	0.210^b^	–0.235^b^	0.460^b^	0.154^c^	–0.024
	Nonfan	–0.278^b^	0.115^c^	–0.005	0.084	0.060	0.062	0.537^b^	0.171^c^	0.321^b^	0.019
**2019 (N=299)**											
	Total	–0.043	0.301^b^	0.468^b^	0.183^c^	0.286^b^	0.162^c^	0.136^c^	0.358^b^	0.407^b^	0.143^c^
	Fan	–0.010	0.321^b^	0.516^b^	0.221^b^	0.251^b^	0.151^c^	–0.047	0.423^b^	0.473^b^	0.120^c^
	Nonfan	–0.106	0.174^c^	0.210^b^	0.078	0.248^b^	0.125^c^	0.420^b^	0.119^c^	0.166^c^	0.119^c^
**2020 (N=133)**											
	Total	–0.121	–0.122	0.438^b^	0.206^c^	0.280^c^	0.254^c^	0.258^c^	0.509^b^	0.658^b^	0.289^c^
	Fan	–0.089	–0.042	0.428^b^	0.218^c^	0.325^b^	0.259^c^	0.126	0.547^b^	0.717^b^	0.315^b^
	Nonfan	–0.112	–0.232^c^	0.326^b^	0.152	0.144	0.164	0.414^b^	0.296^c^	0.383^b^	0.159

^a^Organic reach of all contents in the research period (2017-2020).

^b^Highly significant, *P*<.001 (2-tailed).

^c^Significant, *P*<.05 (2-tailed).

### Correlations Between Interactions

We investigated the 1025 social media contents in relation to the Facebook interactions. Table 3 provides the intercorrelations among the 10 types of Facebook interactions. First, the engagement indicators (reactions, shares, comments, clicks) were analyzed with the Spearman correlation method. The first question in this research area was, “How does the direction of correlations change between engagement indicators which express positive and negative emotion?” We found that most of the correlations between engagement indicators had a positive direction; however, negative correlations were observed in some cases, which might be associated with negative emotional characteristics (eg, “sad” and “angry” reactions). Highly significant negative correlations were found between “sad” and “like” reactions (*P*<.001) as well as between “angry” and “like” reactions (*P*<.001). The significant negative correlation between “wow” and “like” reactions (*P*=.013) as well as between “comment” and “like” interactions (*P*<.001) may suggest that Facebook users could express their negative emotions also with the “wow” reaction or a negative emotional comment. By contrast, the significant negative correlations between “comment” and “share” interactions (*P*=.001), and “love” and “share” interactions (*P*=.004) can be explained more by the fan reach than by negative emotions. Social media contents with a high rate of “share” interaction may lead to low fan reach, which can be associated with a decrease in the rate of “love” and “comment” interactions. Overall, negative correlations between engagement indicators can indicate that the integrity of engagement should be assessed cautiously. The next research question was, “How does the strength of positive correlations change between engagement indicators?” Some highly significant correlations were observed between reactions, which indicated different Facebook users’ combined interactions on the same content. The strongest highly significant positive correlations were found between “sad” and “angry” reactions (r_s_=0.302, *P*<.001), “love” and “wow” reactions (r_s_=0.204, *P*<.001), and “love” and “haha” reactions (r_s_=0.141, *P*<.001). Other combinations of interactions on the given content may have arrived from different users or the same Facebook user. In this case, the strongest highly significant positive correlations were found between “comments” and “clicks” (r_s_=0.417, *P*<.001), and between “comments” and reactions, especially “sad” (r_s_=0.196, *P*<.001), “haha” (r_s_=0.174, *P*<.001), and “angry” reactions (r_s_=0.165, *P*<.001). Together, these results provide important insights into the type of combined reactions or interactions applied by Facebook users to react on smoking cessation support contents.

Second, the correlations related to negative Facebook interactions were analyzed. The last question in this research area was, “What is the relationship between engagement indicators and negative Facebook interactions?” We observed 3 correlations between some reactions (“like,” “wow,” and “sad”) and negative Facebook interactions, which might be associated with negative emotional characteristics. A significant negative correlation was observed between the “like” reaction and negative interaction (*P*=.040), which can support its role among the engagement indicators. We found significant positive correlations between the “wow” reaction and negative interactions (*P*=.016), or between the “sad” reaction and negative interactions (*P*=.003), which can question their role among the engagement indicators. Another significant positive correlation was noted between “click” and negative interactions (*P*=.03), which can be explained more by a combination of interactions than by negative emotions. Facebook users might have collected more information by clicks before they applied negative interactions buttons. In summary, these results show that some engagement indicators correlated with negative Facebook interactions, which are activities against the smoking cessation intervention (eg, reports of spam or unlike of page).

**Table 3:** Spearman correlation matrix for the comparison between Facebook interactions regarding 1025 social media contents (N=1025).

			Reaction													Share			Comment			Click			Negative interactions

			Like		Love		Haha		Wow		Sad		Angry												
**Reaction**																									
	Like	1.00		0.008		0.063^a^		–0.077^a^		–0.120^b^		–0.136^b^		0.094^a^			–0.130^b^			0.147^b^			–0.064^a^		
	Love			1.00		0.141^b^		0.204^b^		0.063^a^		0.008		–0.090^a^			0.123^b^			–0.007			0.009		
	Haha					1.00		0.132^b^		0.140^b^		0.080^a^		–0.049			0.174^b^			0.002			0.052		
	Wow							1.00		0.104^a^		0.122^b^		–0.055			0.145^b^			0.087^a^			0.076^a^		
	Sad									1.00		0.302^b^		–0.011			0.196^b^			0.070^a^			0.091^a^		
	Angry											1.00		0.004			0.165^b^			0.097^a^			0.032		
Share															1.00			–0.105^a^			0.097^a^			0.029	
Comment																		1.00			0.417^b^			0.059	
Click																					1.00			0.066^a^	
Negative interactions																								1.00	

^a^Significant, *P*<.05 (2-tailed).

^b^Highly significant, *P*<.001 (2-tailed).

## Discussion

### Principal Results

The first aim in this research was to understand the association between organic reach and Facebook interactions during a smoking cessation intervention. Findings suggest that total organic reach may correlate positively with most Facebook interactions, except the “like” and “share” interactions. The highly significant negative correlation between total organic reach and the “like” reaction can highlight the widespread misconception of “more likes cause higher reach” [2]. The lack of a significant relationship between total organic reach and the “share” interaction may be explained by the unique function of “share.” “Share” is the only interaction that has a direct effect on organic reach by sending the given content to others [24-26]. In this study, the “share” interaction correlated positively with nonfan reach, and negatively with fan reach. This positive correlation suggests that Facebook users who applied the “share” button usually sent the content to nonfan Facebook users, which led to higher nonfan reach. By contrast, the significant negative correlation between “shares” and fan reach highlights that the Facebook algorithm might decrease the fan reach in response to the notable increase in nonfan reach. Therefore, the “share” interaction may have a higher impact on content algorithmic ranking than the previous “page like,” which is the basic requirement of fan reach. This mechanism can act as a “brake” of viral reach. Previous studies have showed that fan Facebook users are characterized by higher “share” activity than nonfans [2]. If “shares” would correlate positively with fan reach, a higher “share” activity of fan users could lead to a viral reach. This might be a possible explanation of the negative correlation between “shares” and nonfan reach as a “brake effect.”

By contrast, the significant positive correlation between “clicks” and nonfan reach may be explained by an opposite cause–effect relationship. Nonfan Facebook users might apply “clicks” more often to gain new information than fan users. Nevertheless, the significant positive correlations between organic reach and other interactions can provide important insights into how to enhance total organic reach, especially fan reach. The “Haha” reaction, “comments” and the “love” reaction proved the most typical fan-specific interactions, which could also achieve the highest increase in total organic reach. Finally, an unanticipated finding was the significant positive correlation between organic reach and negative Facebook interactions, probably due to the fact that Facebook users could express their aversions related to the given content with negative interactions (eg, post hides, or reports of spam).

The second objective of the research was to assess the correlations between positive and negative emotional interactions during a Facebook-based smoking cessation intervention. Negative emotional reactions (“wow,” “sad,” “angry” reactions) and comments were sharply separated from the other engagement indicators. First, significant negative correlations were observed between the “like” reaction and “wow,” “sad,” “angry” reactions, and comments. Second, we found that a high rate of “wow” or “sad” reactions was associated with negative Facebook interactions. These results suggests that Facebook users could express their negative emotions with these interactions as a resistance to smoking cessation.

Finally, some special combinations of interactions are discussed. The correlations of the “share” interaction should be interpreted considering its special impact on fan reach. “Share” interactions may indirectly reduce fan reach and the rate of fan-specific interactions. This effect of “shares” can explain the significant negative correlations with “love” and “comment” interactions. In addition, the lack of a significant relationship with “haha,” “wow,” “sad,” and “angry” reactions can be explained by the consequential low fan reach. The significant positive correlations of “shares” with “like” and “click” interactions might indicate frequently used combinations. Analyzing our data, the significant positive correlation between “click” and negative interactions was also a thought-provoking combined interaction, which may help to elucidate the reason why negative Facebook interactions could increase the organic reach.

### Hypotheses for Future Research

This was an exploratory, hypothesis-generating study to get insights into the correlations of organic reach, engagement, and Facebook users’ interactions. Textbox 3 presents some recommended hypotheses for more rigorous testing in the future. First, the hypotheses related to organic reach are discussed. We assume that the “like” reaction is likely to decrease the organic reach of smoking cessation support contents on a public Facebook page. Facebook users often used this interaction [2,11], perhaps this is why the Facebook algorithm may use it as a negative element in calculating organic reach. Future studies investigating the “like” reaction would be very interesting in Facebook-based smoking cessation interventions or other public health campaigns. It is also hypothesized that “share” interactions can increase the nonfan reach, and decrease the fan reach of smoking cessation support contents on a public Facebook page. This finding may have important implications for designing social media contents for a target population. In order to reach new (nonfan) Facebook users, “share” interactions on the given content should be encouraged. However, these social media contents probably fail to reach fan Facebook users according to our hypothesis. Further research to analyze the role of “shares” in fan reach and nonfan reach could provide more definitive evidence.

It is assumed that the fan reach would be increased by “haha” and “love” reactions and “comments,” which would have the highest impact on the total organic reach of smoking cessation support contents on a public Facebook page. This finding may be a useful tool when creating Facebook posts to reach fan users during smoking cessation interventions or other public health campaigns. However, public health professionals who make Facebook posts should give suggestions for their audience about interactions carefully. Some techniques to increase engagement (called “engagement bait”) should be avoided, because if Facebook detects this, it will automatically reduce the organic reach. In future investigations, it might be advantageous to use various contents to give suggestions with and without “engagement bait” for the Facebook page’s audience about “like,” “haha,” “love” reactions, “shares,” and “comments.” It should be also noted that organic reach and interactions data for future studies are easily accessible in “Facebook Insights” for 6 months, or in the “Post Details” for more than 6 months. Further studies, which analyze these annual data of different public health Facebook pages, may help us to understand how the Facebook algorithm is modified each year, and public health interventions can follow these changes in the future. Facebook interactions are just one way of trying to improve organic reach of the given content in a public Facebook page. Future research should also consider other elements of the Facebook algorithm (eg, the timing of published content) and other parallel used deliveries (eg, Facebook groups, Facebook stories) [29].

Second, the hypotheses related to engagement indicators are discussed. An important issue for future research is to find which interactions can be classified as engagement indicators on Facebook [33]. Based on this study, it is hypothesized that negative Facebook interactions, negative emotional comments, and reactions (“wow,” “sad,” “angry”) would reduce the engagement of Facebook-based smoking cessation interventions, because these interactions were separated from the other engagement indicators owing to the opposite direction of correlation. Furthermore, these results suggest that “wow” should not be considered a positive reaction, as it seems to have more similarities to a negative emotional response. It is also assumed that “like,” “love,” “haha” reactions, shares, positive comments, and clicks would raise the engagement of Facebook-based smoking cessation interventions. Future investigations should assess the impact of these Facebook interactions on other dimensions of engagement (eg, the amount or duration of usage) [29,33]. More broadly, research is also needed to determine the role of these interactions in other Facebook-based public health campaigns with different aims. For example, the “sad” reaction or negative emotional comment in addictology may indicate a resistance to the smoking cessation intervention, whereas the same interactions in cancer prevention may express Facebook users’ engagement as a response to stories by patients with cancer [36].

Lastly, our findings suggest that some combined interactions (eg, “comments” and “clicks”) would increase the probability of eliciting the other interaction (eg, a high number of “comments” can associate with the rise of “clicks”). This result is only relevant for Facebook-based smoking cessation interventions. In future investigations, self-report questionnaires might be used to explore the popular interactions of different public health target groups [29]. Despite its exploratory nature, this study can improve the practical implementation of these combined interactions. For example, if a public health professional wants to increase the rate of “clicks” (eg, to selecting a website), the usage of social media contents which generate “comments” may be more advantageous than the usage of contents which evoke reactions. This can be also an important issue for future research.

**Hypotheses for future testing based on this research.**

**Organic reach.** **Hypotheses for future testing are:**

- “Like” reactions would decrease the organic reach of smoking cessation support contents on a public Facebook page.
- “Share” interactions would significantly increase the nonfan reach, and decrease the fan reach of smoking cessation support contents on a public Facebook page.
- “Haha,” “love” reaction, and “comments” would have the highest impact on fan reach, and total organic reach of smoking cessation support contents on a public Facebook page.

**Engagement indicators. Hypotheses for future testing are:**

- Negative Facebook interactions, negative emotional comments, and reactions (“wow,” “sad,” “angry”) would reduce the engagement of Facebook-based smoking cessation interventions.
- “Like,” “love,” “haha” reactions, shares, positive comments, and clicks would raise the engagement of Facebook-based smoking cessation interventions.
- Some combined interactions (eg, “comments” and “clicks”) would increase the probability of eliciting the other interaction in a Facebook-based smoking cessation intervention.

### Limitations

It should be noted that we used a convenience sample, and the audience of the investigated public Facebook page was heterogeneous. Furthermore, the availability of participants’ demographic data is also limited to a post level because it could not be retrieved data from the “Post Details.” That is why the results of the second correlational analysis cannot be generalized widely. Therefore, the correlations between the Facebook users’ interactions can only be interpreted in the context of a smoking cessation intervention based on motivational interviewing.

However, these limitations might not affect the interpretation of the first correlational analysis because the relationship between the organic reach and Facebook interactions depends only on the “Facebook algorithm of content ranking.” This algorithm is presumably free from demographic data or smoking status. Using age, gender, or other demographic data to reach users are against Facebook Community Standards. Furthermore, smoking status cannot be an element of the Facebook algorithm either because it is not registered in Facebook. Lastly, the Facebook algorithm probably evaluates the performance of the given post, rather than its public health content which supports smoking cessation or not. Therefore, the results of the first correlational analysis between the organic reach and Facebook interactions can be generalized for other Facebook-based interventions which use a public Facebook page. At the same time, these results should be interpreted with caution, regarding the other potential elements of the Facebook algorithmic content ranking (eg, mainly image-type post was included in this current research).

Finally, Facebook advertising can indirectly raise the organic reach and the number of interactions during the advertising period. The paid reach (66,463 people) was about double than organic reach (30,191 people) in the last month of our research period. However, this potential indirect growth in organic reach and the number of interactions affected all Facebook posts equally during the advertising period. Furthermore, boosted (paid) Facebook posts were excluded to avoid the direct effects of advertising.

### Conclusions

The purpose of this research was to understand the relationship between Facebook users’ interactions, organic reach, and engagement, which may be informative for further research and the design of Facebook-based smoking cessation interventions. The key strengths of this study are the large size of the data set and its long duration, which can shine new light on the pattern of change in the Facebook algorithm. Returning to the research questions described at the beginning of this paper, it is now possible to state that most Facebook interactions can correlate with the organic reach of a smoking cessation intervention positively, while the “like” reaction can correlate negatively. The strength of these correlations was proved to be different, which may mean variant emphases in the Facebook algorithm. This is the first study to report a disadvantage of the “like” reaction and highlight the advantages of other interactions (eg, the “haha” reaction or “comments”) in algorithmic content ranking on Facebook. The analysis of fan reach and nonfan reach suggests the need for further categorization of fan-specific interactions (eg, “haha” or “love” reactions) and nonfan-specific interactions (eg, “shares” and “clicks”). The generalizability of these results is wide for Facebook-based public health interventions, because these correlations depend only the Facebook algorithm, which does not contain demographic data, smoking status, or other health risks. In addition, the Facebook algorithm of content ranking calculates with the performance of the given post, and it ignores the specific aim of the given public health intervention or the given theory or strategy of health behavioral change used in the Facebook post. This study made an attempt to explore the relationship between the Facebook algorithm and users’ interactions; nevertheless, further research is needed to investigate other elements of the Facebook algorithm.

The correlational analysis of Facebook interactions raises some exciting hypotheses for future testing. A novel classification of engagement indicators should be considered in Facebook-based smoking cessation interventions. Negative Facebook interactions; negative emotional comments; and “wow,” “sad,” and “angry” reactions may decrease the engagement, whereas “like,” “love,” “haha” reactions, shares, positive comments, and clicks may increase the engagement of these interventions. Furthermore, other specific combinations of interactions can be useful to raise the probability of certain interactions under smoking cessation support contents. Based on our findings, we suggest implementing the continuous evaluation of Facebook interactions during interventions.

## Appendix

Multimedia Appendix 1 Stimuli: examples of smoking cessation support contents based on motivational interviewing (doc).

Multimedia Appendix 2 Exclusion criteria: examples of excluded social media contents (doc).

Multimedia Appendix 3 Original data (xls).

## References

[1] Nabi RL, Prestin A, So J (2013). Facebook friends with (health) benefits? Exploring social network site use and perceptions of social support, stress, and well-being. Cyberpsychol Behav Soc Netw.

[2] Kite J, Foley BC, Grunseit AC, Freeman B (2016). Please Like Me: Facebook and Public Health Communication. PLoS One.

[3] Park H, Rodgers S, Stemmle J (2011). Health Organizations’ Use of Facebook for Health Advertising and Promotion. Journal of Interactive Advertising.

[4] Kuipers M, Beard E, West R, Brown J (2018). Associations between tobacco control mass media campaign expenditure and smoking prevalence and quitting in England: a time series analysis. Tob Control.

[5] Durkin S, Brennan E, Wakefield M (2012). Mass media campaigns to promote smoking cessation among adults: an integrative review. Tob Control.

[6] Brown J, Kotz D, Michie S, Stapleton J, Walmsley M, West R (2014). How effective and cost-effective was the national mass media smoking cessation campaign ‘Stoptober’?. Drug and Alcohol Dependence.

[7] Xu X, Alexander RL, Simpson SA, Goates S, Nonnemaker JM, Davis KC, McAfee T (2015). A cost-effectiveness analysis of the first federally funded antismoking campaign. Am J Prev Med.

[8] Allom V, Jongenelis M, Slevin T, Keightley S, Phillips F, Beasley S, Pettigrew S (2018). Comparing the Cost-Effectiveness of Campaigns Delivered Various Combinations of Television and Online Media. Front Public Health.

[9] Bold KW, Hanrahan TH, O'Malley SS, Fucito LM (2016). Exploring the Utility of Web-Based Social Media Advertising to Recruit Adult Heavy-Drinking Smokers for Treatment. J Med Internet Res.

[10] Thackeray R, Neiger BL, Smith AK, Van Wagenen SB (2012). Adoption and use of social media among public health departments. BMC Public Health.

[11] Kite J, Grunseit A, Li V, Vineburg J, Berton N, Bauman A, Freeman B (2019). Generating Engagement on the Make Healthy Normal Campaign Facebook Page: Analysis of Facebook Analytics. JMIR Public Health Surveill.

[12] Andrade EL, Evans WD, Barrett N, Edberg MC, Cleary SD (2018). Strategies to Increase Latino Immigrant Youth Engagement in Health Promotion Using Social Media: Mixed-Methods Study. JMIR Public Health Surveill.

[13] Ford K, Albritton T, Dunn T, Crawford K, Neuwirth J, Bull S (2019). Youth Study Recruitment Using Paid Advertising on Instagram, Snapchat, and Facebook: Cross-Sectional Survey Study. JMIR Public Health Surveill.

[14] Barklamb A, Molenaar A, Brennan L, Evans S, Choong J, Herron E, Reid M, McCaffrey T (2020). Learning the Language of Social Media: A Comparison of Engagement Metrics and Social Media Strategies Used by Food and Nutrition-Related Social Media Accounts. Nutrients.

[15] Chan L, O'Hara B, Phongsavan P, Bauman A, Freeman B (2020). Review of Evaluation Metrics Used in Digital and Traditional Tobacco Control Campaigns. J Med Internet Res.

[16] Theiss SK, Burke RM, Cory JL, Fairley TL (2016). Getting Beyond Impressions: An Evaluation of Engagement with Breast Cancer-related Facebook Content. Mhealth.

[17] Neiger BL, Thackeray R, Van Wagenen SA, Hanson CL, West JH, Barnes MD, Fagen MC (2012). Use of social media in health promotion: purposes, key performance indicators, and evaluation metrics. Health Promot Pract.

[18] Hefler M, Kerrigan V, Grunseit A, Freeman B, Kite J, Thomas D (2020). Facebook-Based Social Marketing to Reduce Smoking in Australia's First Nations Communities: An Analysis of Reach, Shares, and Likes. J Med Internet Res.

[19] Campbell I, Rudan I (2020). Analysis of public engagement with ten major global health topics on a social network profile and a newspaper website. J Glob Health.

[20] Merchant G, Weibel N, Patrick K, Fowler JH, Norman GJ, Gupta A, Servetas C, Calfas K, Raste K, Pina L, Donohue M, Griswold WG, Marshall S (2014). Click "like" to change your behavior: a mixed methods study of college students' exposure to and engagement with Facebook content designed for weight loss. J Med Internet Res.

[21] Rus HM, Cameron LD (2016). Health Communication in Social Media: Message Features Predicting User Engagement on Diabetes-Related Facebook Pages. Ann Behav Med.

[22] Lee Y, Phua J, Wu T (2020). Marketing a health Brand on Facebook: Effects of reaction icons and user comments on brand attitude, trust, purchase intention, and eWOM intention. Health Mark Q.

[23] Kim SJ, Marsch LA, Brunette MF, Dallery J (2017). Harnessing Facebook for Smoking Reduction and Cessation Interventions: Facebook User Engagement and Social Support Predict Smoking Reduction. J Med Internet Res.

[24] Zhang N, Tsark J, Campo S, Teti M (2015). Facebook for Health Promotion: Female College Students' Perspectives on Sharing HPV Vaccine Information Through Facebook. Hawaii J Med Public Health.

[25] Obamiro K, West S, Lee S (2020). Like, comment, tag, share: Facebook interactions in health research. Int J Med Inform.

[26] Buttle F, Groeger L (2017). Who says what to whom in what channel? A rules theoretic perspective on word-of-mouth marketing. Journal of Marketing Management.

[27] Struik LL, Baskerville NB (2014). The role of Facebook in Crush the Crave, a mobile- and social media-based smoking cessation intervention: qualitative framework analysis of posts. J Med Internet Res.

[28] Bennetts SK, Hokke S, Crawford S, Hackworth NJ, Leach LS, Nguyen C, Nicholson JM, Cooklin AR (2019). Using Paid and Free Facebook Methods to Recruit Australian Parents to an Online Survey: An Evaluation. J Med Internet Res.

[29] Perski O, Blandford A, West R, Michie S (2017). Conceptualising engagement with digital behaviour change interventions: a systematic review using principles from critical interpretive synthesis. Transl Behav Med.

[30] Danaher BG, Boles SM, Akers L, Gordon JS, Severson HH (2006). Defining participant exposure measures in Web-based health behavior change programs. J Med Internet Res.

[31] Couper MP, Alexander GL, Zhang N, Little RJA, Maddy N, Nowak MA, McClure JB, Calvi JJ, Rolnick SJ, Stopponi MA, Cole JC (2010). Engagement and retention: measuring breadth and depth of participant use of an online intervention. J Med Internet Res.

[32] Cole-Lewis H, Perotte A, Galica K, Dreyer L, Griffith C, Schwarz M, Yun C, Patrick H, Coa K, Augustson E (2016). Social Network Behavior and Engagement Within a Smoking Cessation Facebook Page. J Med Internet Res.

[33] Platt T, Platt J, Thiel DB, Kardia SLR (2016). Facebook Advertising Across an Engagement Spectrum: A Case Example for Public Health Communication. JMIR Public Health Surveill.

[34] CigiSzünet.

[35] Pócs D, Kovács R, Óvári T, Erdős C, Kelemen O (2019). [Tobacco reduction on Facebook among 14-35-year-olds]. Orv Hetil.

[36] Tang C, Zhou L, Plasek J, Rozenblum R, Bates D (2017). Comment Topic Evolution on a Cancer Institution’s Facebook Page. Appl Clin Inform.

