# Supplementary material for: Facebook Users’ Interactions, Organic Reach, and Engagement in a Smoking Cessation Intervention: Content Analysis
Source: J Med Internet Res. 2021 Jun 21;23(6):e27853. doi: 10.2196/27853 (PMC8277334; doi:10.2196/27853)

# Multimedia appendix 1

## Stimuli: examples of smoking cessation support contents based on motivational interviewing.


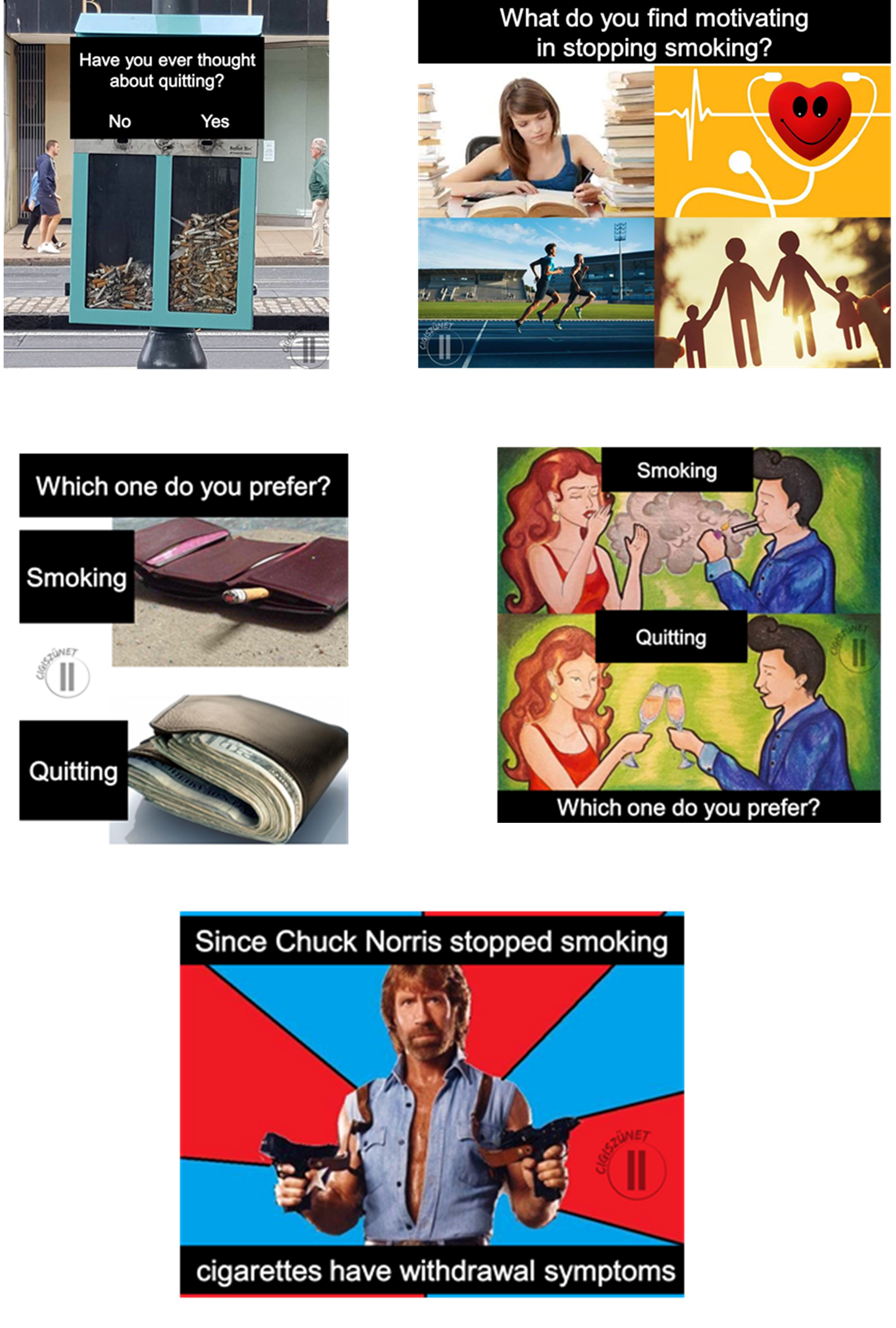


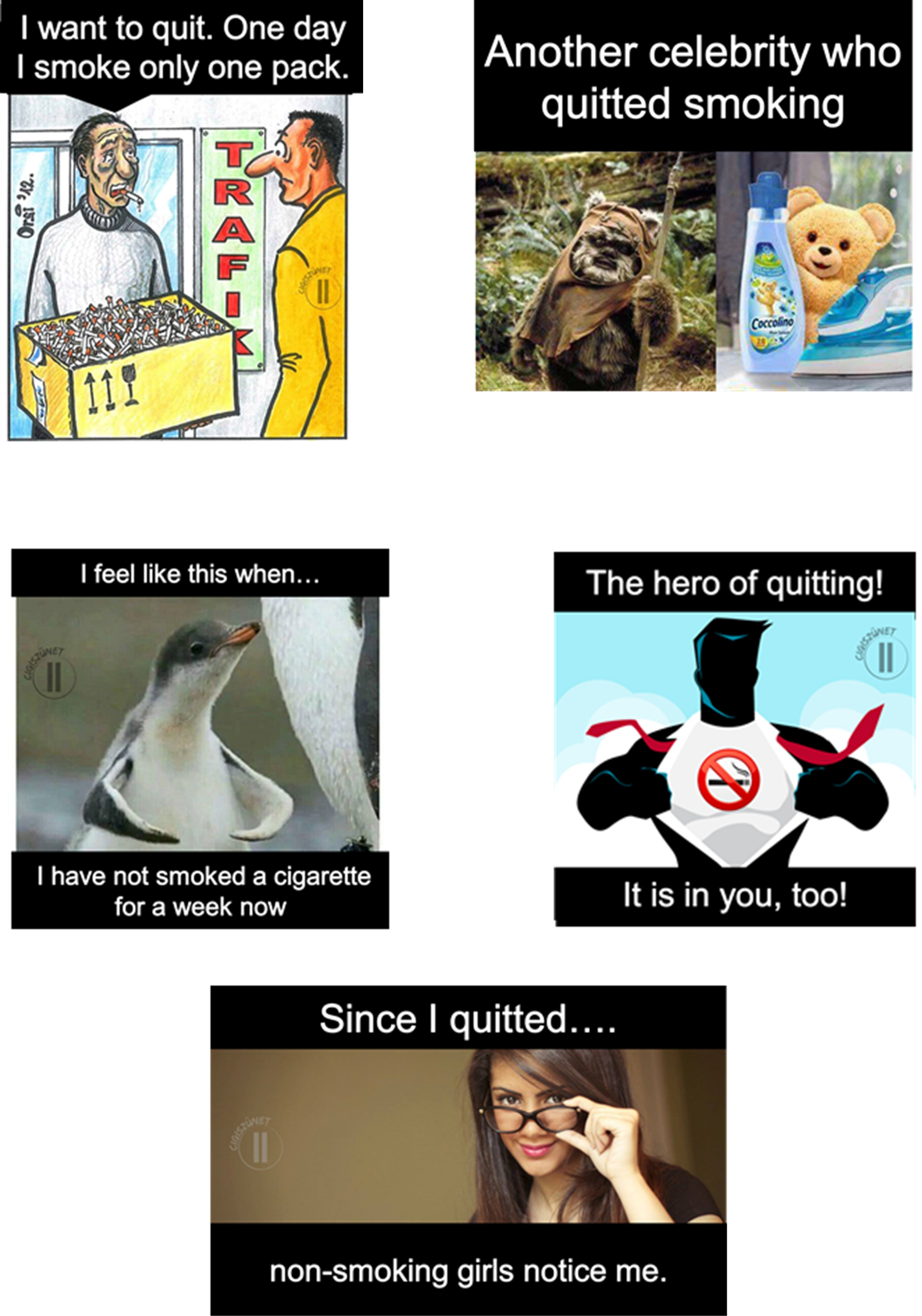


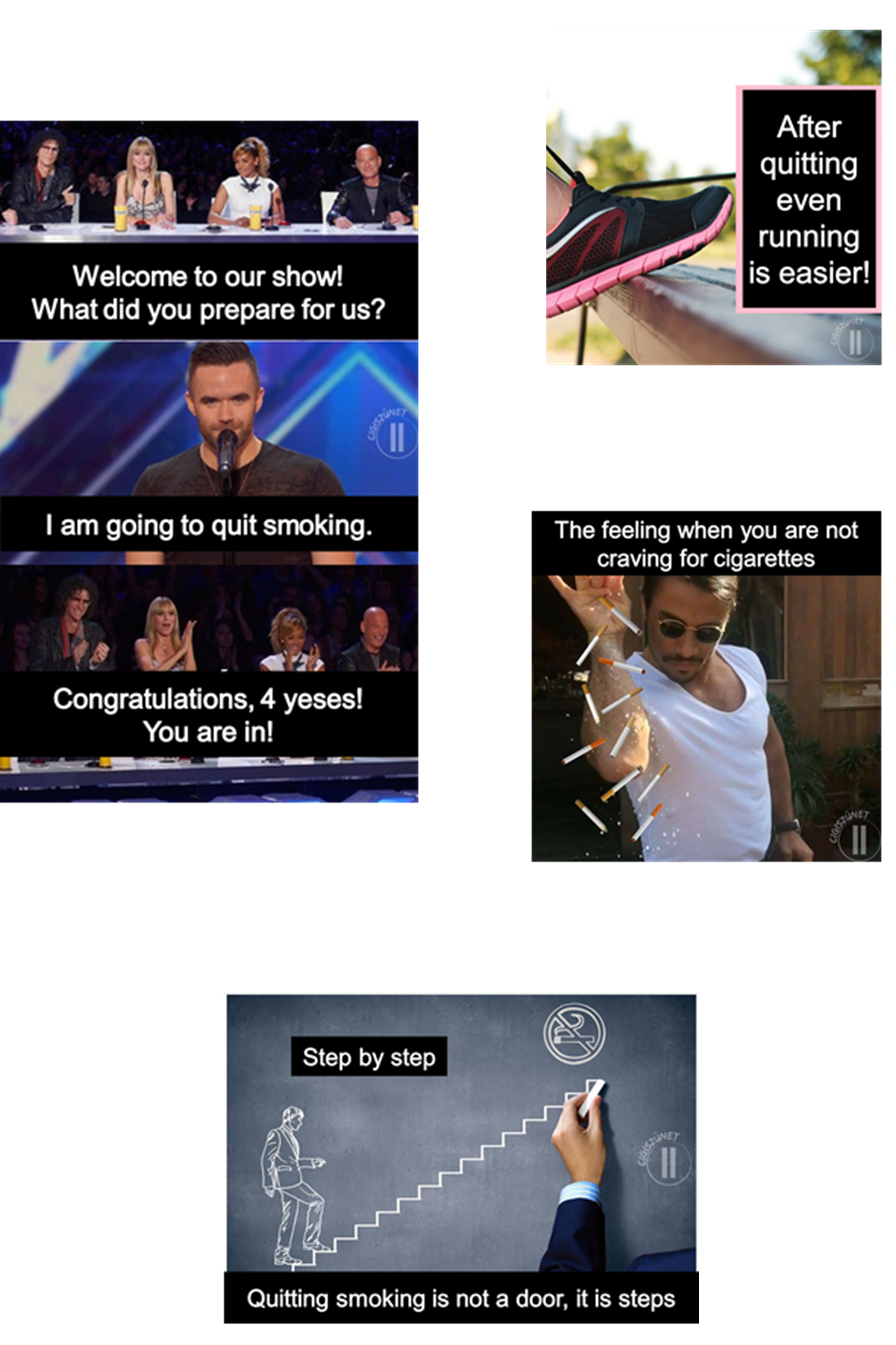


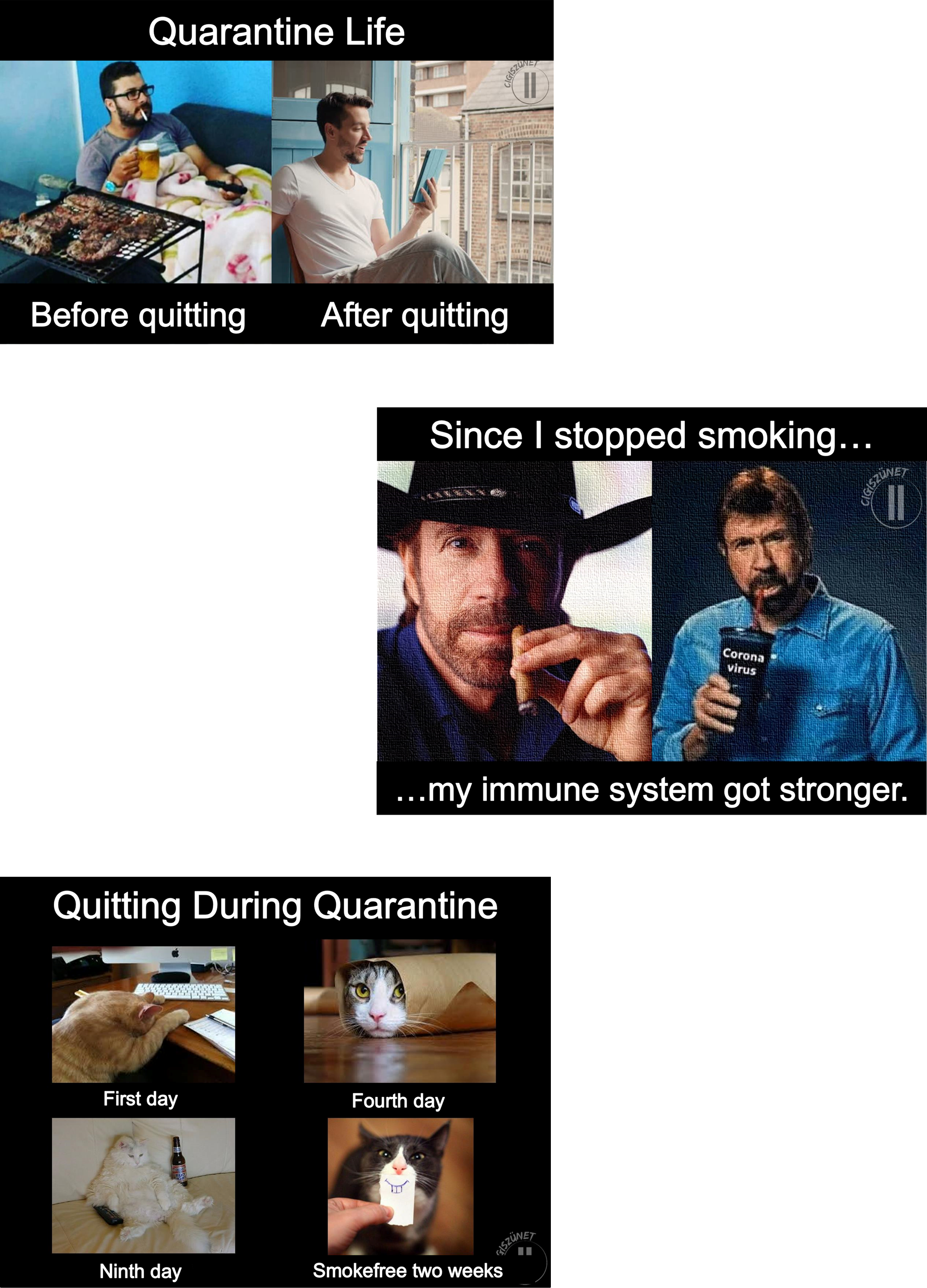

Supplement: Multimedia Appendix 1 [file jmir_v23i6e27853_app1.docx]
