# Supplementary material for: Facebook Users’ Interactions, Organic Reach, and Engagement in a Smoking Cessation Intervention: Content Analysis
Source: J Med Internet Res. 2021 Jun 21;23(6):e27853. doi: 10.2196/27853 (PMC8277334; doi:10.2196/27853)

# Multimedia appendix 2

## Exclusion criteria: examples of excluded social media contents.

### Boosted Facebook posts.

These contents were promoted by paid Facebook advertising after publication to increase reach and engagement.

### Facebook videos and other video contents.

Their interaction data are basically different from image posts (e.g., minutes viewed, 10-second views), and Facebook algorithm evaluates these contents different during content ranking.

### Administrator's Facebook posts.

These contents did not address to smokers directly.


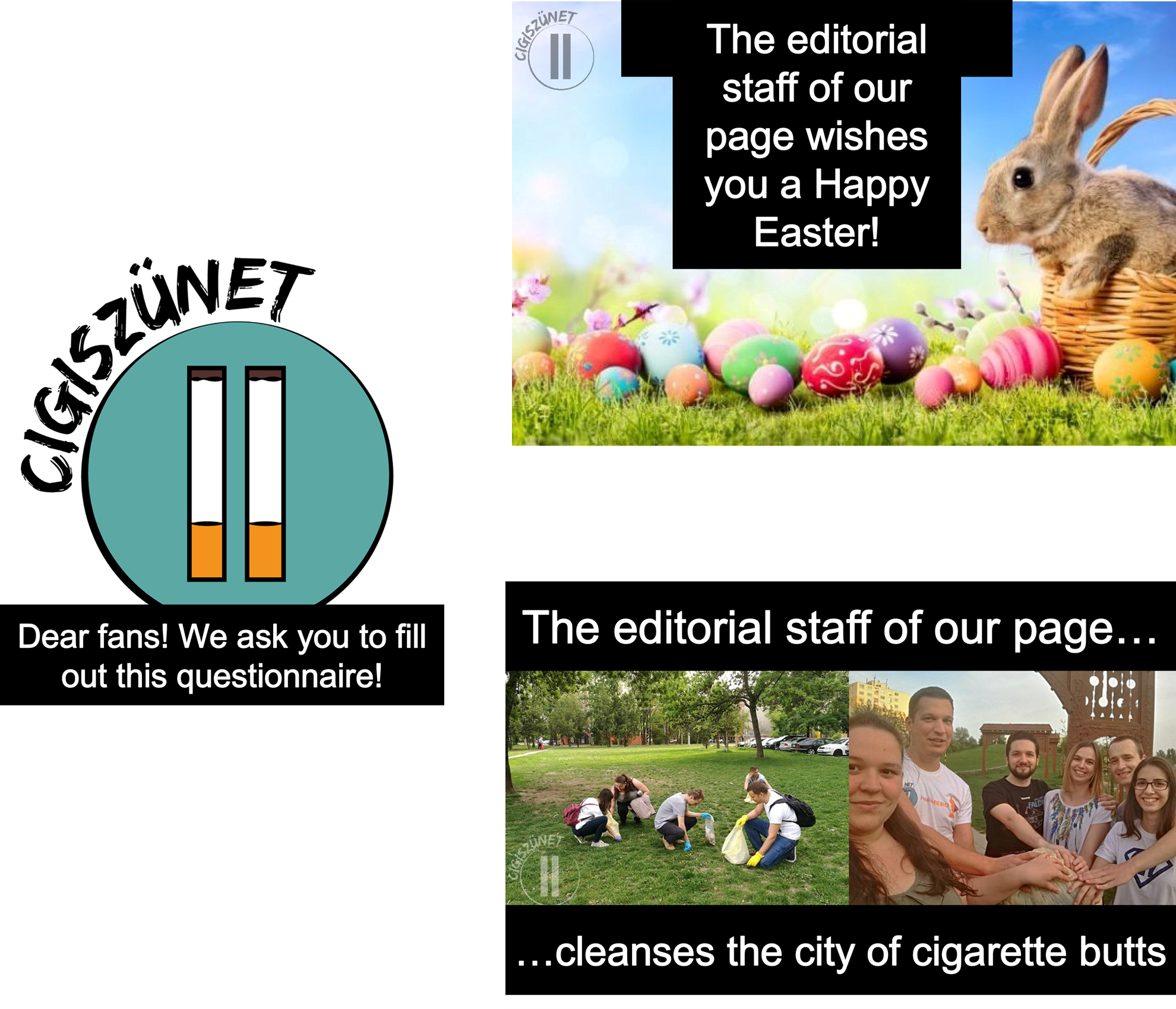


### Facebook posts which were targeted at non-smokers.

These contents did not address to smokers directly.


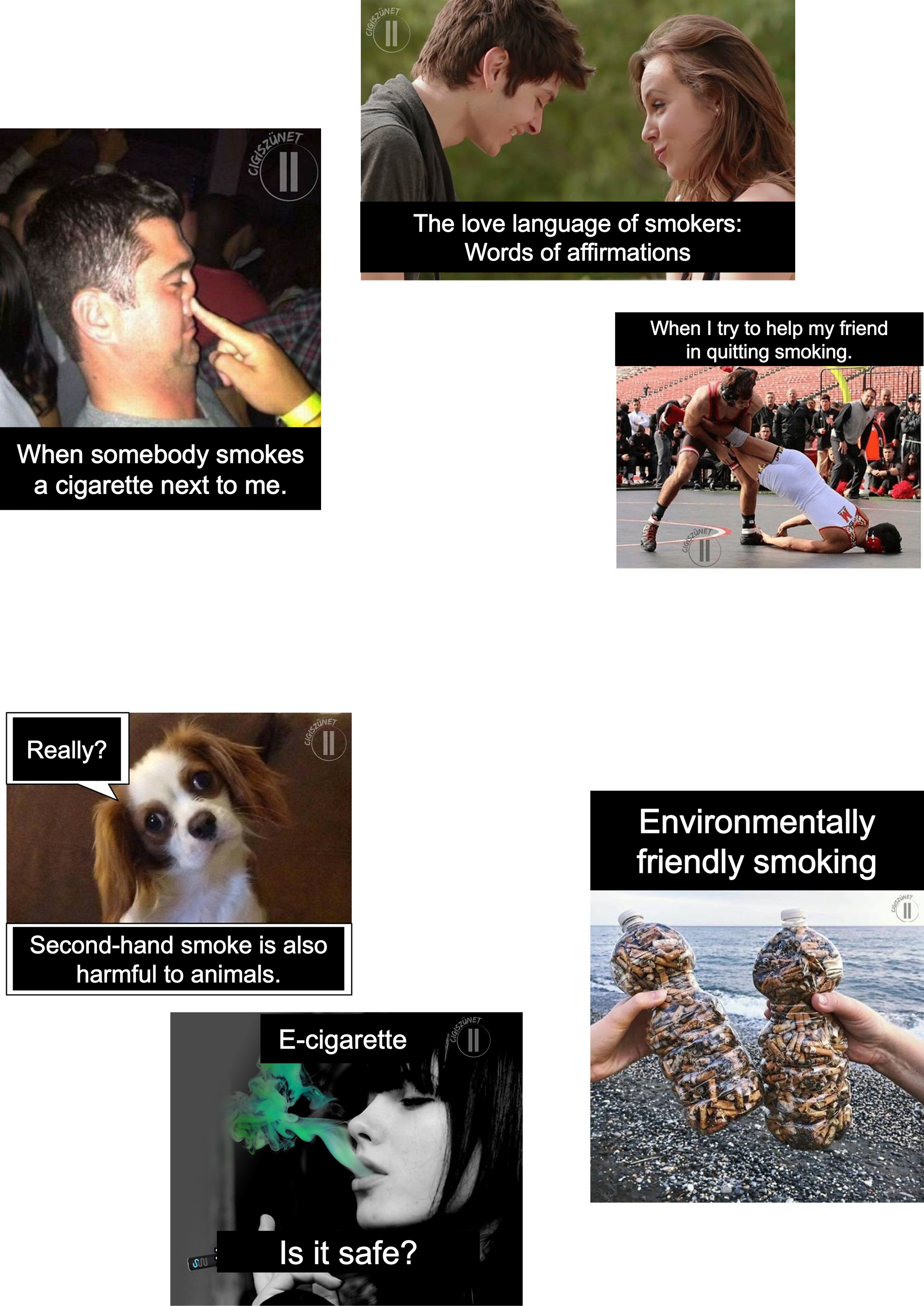


### Facebook posts with non-cessation topic.

These contents did not support smoking cessation directly.

### Motivational interviewing non-adherent Facebook posts.

These contents did not conform perfectly to the spirit of motivational interviewing during the retrospective analysis.


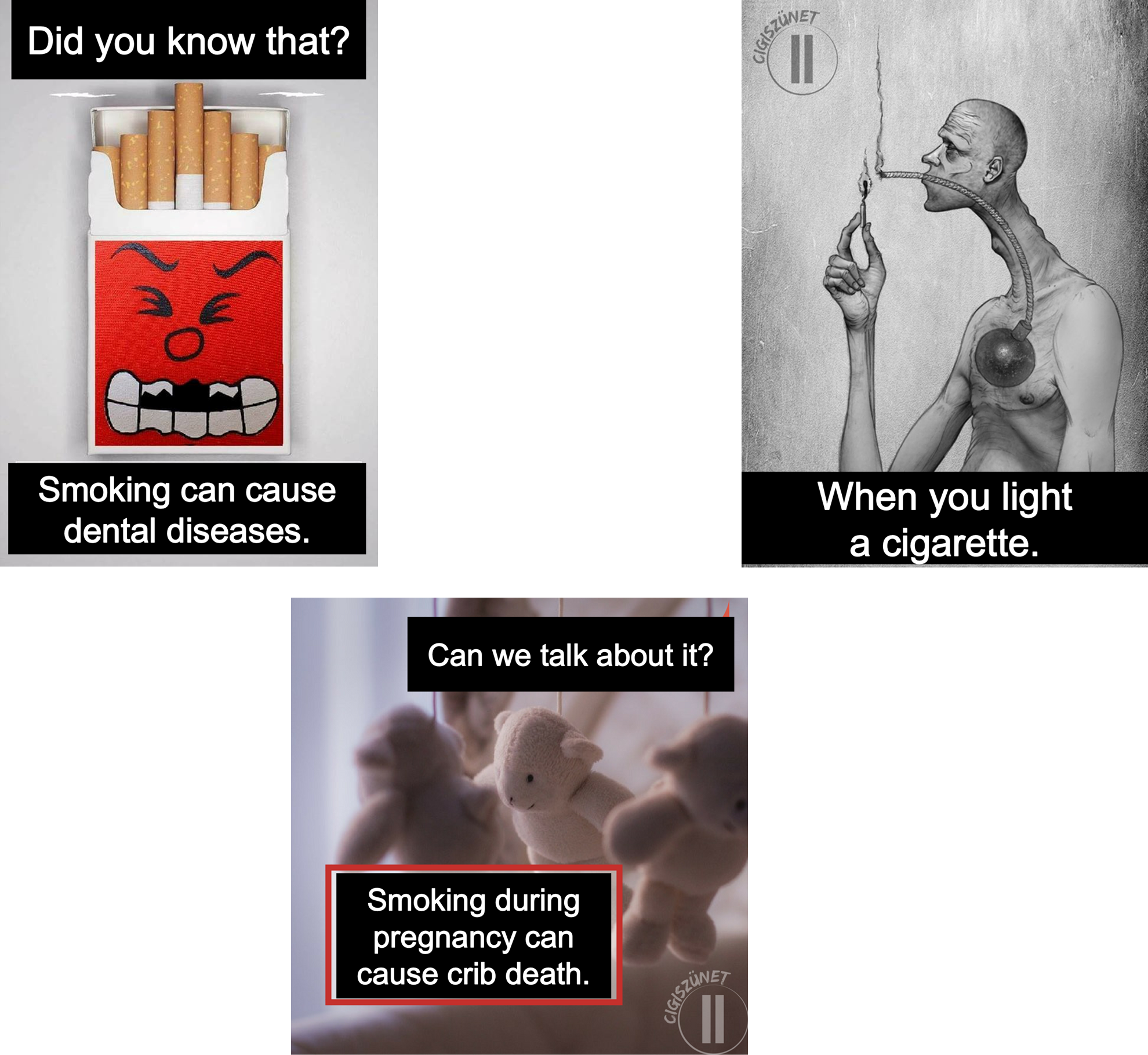

Supplement: Multimedia Appendix 2 [file jmir_v23i6e27853_app2.docx]
